# PDON: Parkinson’s disease ontology for representation and modeling of the Parkinson’s disease knowledge domain

**DOI:** 10.1186/s12976-015-0017-y

**Published:** 2015-09-22

**Authors:** Erfan Younesi, Ashutosh Malhotra, Michaela Gündel, Phil Scordis, Alpha Tom Kodamullil, Matt Page, Bernd Müller, Stephan Springstubbe, Ullrich Wüllner, Dieter Scheller, Martin Hofmann-Apitius

**Affiliations:** Department of Bionformatics, Fraunhofer Institute for Algorithms and Scientific Computing, 53754 Sankt Augustin, Germany; Rheinische Friedrich-Wilhelms-Universität Bonn, Bonn-Aachen International Center for IT, 53113 Bonn, Germany; Department of Neurology, University of Bonn, 53105 Bonn, Germany; Informatics group, UCB Pharma, 208 Bath Road, Slough, UK; Pharmacology Parkinson′s Disease and Movement Disorders, UCB Pharma S.A., Chemin du Foriest, B-1420 Braine-l’Allued, Belgium

**Keywords:** Parkinson’s disease, ontology, disease modeling, knowledge engineering

## Abstract

**Background:**

Despite the unprecedented and increasing amount of data, relatively little progress has been made in molecular characterization of mechanisms underlying Parkinson’s disease. In the area of Parkinson’s research, there is a pressing need to integrate various pieces of information into a meaningful context of presumed disease mechanism(s). Disease ontologies provide a novel means for organizing, integrating, and standardizing the knowledge domains specific to disease in a compact, formalized and computer-readable form and serve as a reference for knowledge exchange or systems modeling of disease mechanism.

**Methods:**

The Parkinson’s disease ontology was built according to the life cycle of ontology building. Structural, functional, and expert evaluation of the ontology was performed to ensure the quality and usability of the ontology. A novelty metric has been introduced to measure the gain of new knowledge using the ontology. Finally, a cause-and-effect model was built around PINK1 and two gene expression studies from the Gene Expression Omnibus database were re-annotated to demonstrate the usability of the ontology.

**Results:**

The Parkinson’s disease ontology with a subclass-based taxonomic hierarchy covers the broad spectrum of major biomedical concepts from molecular to clinical features of the disease, and also reflects different views on disease features held by molecular biologists, clinicians and drug developers. The current version of the ontology contains 632 concepts, which are organized under nine views. The structural evaluation showed the balanced dispersion of concept classes throughout the ontology. The functional evaluation demonstrated that the ontology-driven literature search could gain novel knowledge not present in the reference Parkinson’s knowledge map. The ontology was able to answer specific questions related to Parkinson’s when evaluated by experts. Finally, the added value of the Parkinson’s disease ontology is demonstrated by ontology-driven modeling of PINK1 and re-annotation of gene expression datasets relevant to Parkinson’s disease.

**Conclusions:**

Parkinson’s disease ontology delivers the knowledge domain of Parkinson’s disease in a compact, computer-readable form, which can be further edited and enriched by the scientific community and also to be used to construct, represent and automatically extend Parkinson’s-related computable models. A practical version of the Parkinson’s disease ontology for browsing and editing can be publicly accessed at http://bioportal.bioontology.org/ontologies/PDON.

**Electronic supplementary material:**

The online version of this article (doi:10.1186/s12976-015-0017-y) contains supplementary material, which is available to authorized users.

## Background

Parkinson’s disease (PD), a progressive movement disorder, is the second most common neurodegenerative disease [1]. The molecular etiology of sporadic PD has not been resolved yet and therefore PD is often called an “idiopathic” disease. In recent years, several attempts at elucidating the molecular etiology of PD have generated large omics data sets [2]. The emerging systems view on the pathology of neurodegenerative diseases (NDDs) requires an efficient strategy to aggregate, standardize, represent, and communicate biomedical information through controlled vocabularies and ontologies [3]. An ontology is defined as “an explicit specification of a conceptualization”, which aims to facilitate knowledge sharing [4].

Ontologies are the basis for automated reasoning [5], for large-scale annotation of entire genomes [6, 7], for data mining in microarray data [8], for prediction of biomolecular interactions [9], and for semantic and ontological search in poorly structured information sources [10, 11].

A large portfolio of widely accepted and widely used ontologies including Gene Ontology [7], the Sequence Ontology [12] and the Microarray Gene Expression Database Ontology [8] has evolved in the life sciences. Gene ontology (GO) is the most frequently used ontology in biomedical sciences, which provides standard functional annotations for genes and gene products. Although GO has facilitated understanding of high-throughput results by means of enrichment analysis, one of its significant limitations is that it does not capture domain-specific biological complexity [13]. For example, GO is devoid of any disease-specific context. It can not be used for answering questions like “which disease subtypes or syndromes are over-represented in my gene or protein set?” Hence, a more useful GO ideally should contain: i. disease-specific annotations, ii. disease-specific categories, and iii. semantics that cover disease knowledge domains.

To include disease-specific biological processes, functionalities, and categories, disease-specific ontologies that cover a broad spectrum of relevant knowledge are required. Disease ontologies may reference source terminologies and vocabularies with a hierarchical concept classification such as the SNOMED CT nomenclature [14], the ICD ontology [15] and the human disease ontology [16]. These ontologies contain human disease concepts but their high-level, broad coverage and the lack of depth in these ontologies restrict their usage for specific disease domains. Malhotra and colleagues addressed this issue in the area of NDDs by construction of Alzheimer’s disease ontology (ADO) to cover clinical, etiological, molecular and cellular mechanism aspects of AD [17]. The aim of developing ADO was to enable semantic mining of patient records and literature for effective retrieval and extraction of accurate AD-related information, which could be used for modeling disease processes.

Similarly, there is a need for organizing the knowledge domain of PD. In response to this unmet need, we aimed at creating a disease ontology for PD (PDON) that spans from the molecular biology of the disease to clinical readouts. PDON has been created with a subclass-based hierarchy that – for the majority of concepts - uses subsumption relation (i.e. is_a). However, based on demand from biomedical experts for richer relations, partonomy relation (i.e. part_of) was also introduced, as a concession to better usability of the ontology by interdisciplinary experts. We demonstrate the ontology's usability through our use cases, keeping in mind that, re-usability by other teams is an important aspect in ontology construction and its adoption by the community. As a proof of concept, we performed evaluation of PDON performance by measuring its ability to recover pre-existing, expert-curated information from the knowledge space of PD in the literature with the aim of generating novel insights and hypotheses.

The power of the ontology can be applied to several scenarios, e.g. building recommendation systems by mapping drug failure events to mechanisms and stages of disease/stages of drug discovery, or distinguishing proven facts from hypotheses and speculations [18]. Furthermore, in the emerging era of systems analysis of NDDs, such a knowledge-driven approach is expected to support the integration of multiscale and multilevel information across different biological scales, from molecular networks to clinical readouts.

To this end, we have proposed a model-driven approach to integrating biomedical knowledge and data into mechanistic models that represent cause-and-effect relationships among molecular entities, biological processes and their corresponding clinical outcomes. Using this strategy in the current study, we demonstrate how PDON can be utilized not only to causally link molecular etiology of PD to impaired biological processes and their corresponding disease outcome (Application scenario 1) but also to annotate experimental datasets with their corresponding knowledge description for further integration into disease models (Application scenario 2).

## Results

**Fig. 1:**
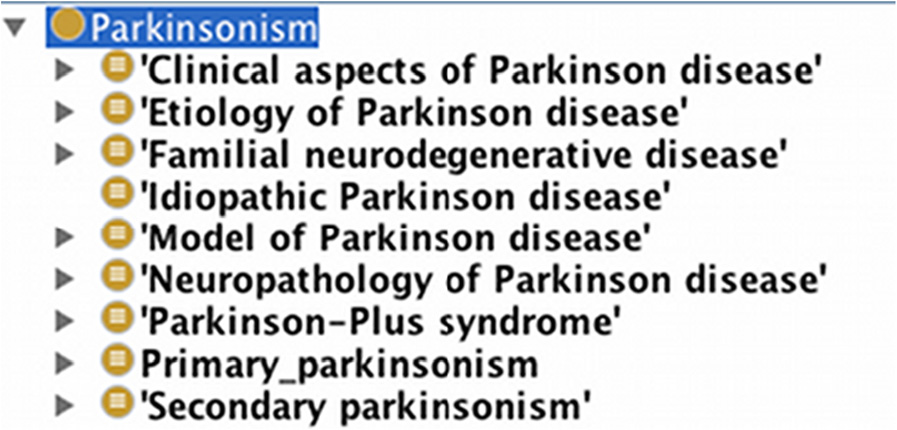
Upper-level classes of PDON as represented in the Protégé ontology editor software. Super-classes represent different biological views (perspectives) suggested by experts under which PD-specific knowledge is modeled

The purpose of the PDON is to communicate and share the PD knowledge in a standard form and support text-mining and knowledge discovery. Furthermore, for the construction of a large, integrative knowledge base on neurodegenerative diseases, PDON can be used for metadata annotation of various omics data sets available in the public domain. The PDON encompasses clinical and non-clinical aspects of PD and is expected to support retrieval of information on syndromes, etiology, treatment, experimental models, diagnosis and symptoms of PD (Fig. 1).

PDON represents a range of key concepts specific to the knowledge domain of Parkinson’s disease through different views, which have been integrated in the ontology. Views are root super-classes that organize concepts within a certain knowledge domain, as they are realized and seen by experts in reality. PDON is represented by nine views:

The view ‘Clinical aspects’ describes a broad range of motor and non-motor features displayed by PD patients. These features have been classified into three upper-level categories that capture clinical concepts related to “diagnostics”, “symptomatology” and “treatment” of PD.

The view ‘Etiology’ captures both genetic and environmental factors that are known to cause familiar PD and Parkinsonism due to toxic dopaminergic cell death, respectively. Toxic and infectious agents as well as genetic mutations are classified within this view; in addition, epidemiologically confirmed factors such as smoking and pesticides have been also included.

The view ‘Model of Parkinson’s disease’ contains various *in-vivo* and *in-vitro* disease models that are in routine use in PD research.

The view ‘Neuropathology’ was included to highlight two prototypic hypotheses of PD-related mechanisms, namely synucleopathy and the emerging tauopathy. It is expected that this view is populated further and enriched with more neuropathological concepts by the PD research community.

The view ‘Familial neurodegenerative disease’ includes those hereditary disorders that are clinically associated with PD, such as Huntington’s and Wilson’s diseases.

‘Idiopathic Parkinson’s disease’, ‘Primary parkinsonism’, ‘Secondary parkinsonism’, and ‘Parkinson-plus Syndrome’ represent four separate views as per recommendation of the clinical expert panel. These views provide a categorized overview on distinct syndromes associated with PD based on their origin of cause. For example, the primary parkinsonism class represents parkinsonian syndromes for which a definite cause has been identified (e.g. mutations in PARK genes), whereas secondary parkinsonism syndromes are induced by a hypothetical cause that is potentially identifiable. Those syndromes with unknown causative factor have been clinically assigned to the Parkinson-plus view.

**Fig. 2:**
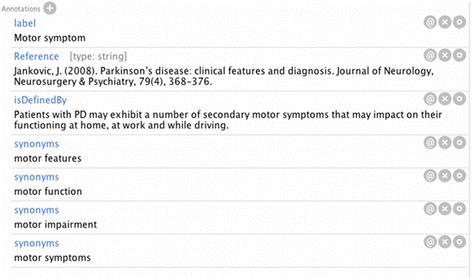
A snapshot of the annotation field for PDON concepts as presented in the Protégé ontology editor. Each PDON concept has been annotated with definition, reference, and synonyms

In PDON, each concept class is supported by a scientific definition, a valid scientific reference (if available) and existing synonyms (Fig. 2). Definitions have been selected from review papers, journal articles and handbooks with consideration of the consensus definitions accepted in the PD research community. It is noteworthy that the PDON is expected to grow over time by inclusion of missing or emerging concepts. Due to dynamic research in the PD field, the structure of ontology is subject to change. We do explicitly invite experts in the field to critically review, revise and optimize the draft ontology presented in this manuscript. The ontology will be updated based on the feedback collected from experts, which includes concept edition, re-defining concepts with missing or insufficient explanation, or new relationship proposal. This is accomplished through the possibility of adding comments or proposals to the ontology’s webpage on the BioPortal repository. The Bioinformatics group at Fraunhofer Institute SCAI that owns the ontology collects these feedbacks and manages the updated releases. The ontology can be freely accessed and downloaded at http://www.scai.fraunhofer.de/en/business-research-areas/bioinformatics/downloads.html.

### Structural evaluation:

PDON was evaluated for structural features reflecting its topology and logical properties. The high-level semantic framework of PDON contains nine super-classes, followed by sub-classes that specifically capture the sub-domain knowledge of PD. PDON was characterized for its structural features using parameters that have been summarized in Table 1. As shown in Table 1, PDON covers the knowledge domain of PD using 632 concept classes for which a high number of synonyms has been collected. The depth and width of the ontology reflect sufficient coverage of the PD knowledge domain with a reasonable distribution of concepts at various levels. The so-called Fanout-ness factor represents distribution of concepts over the entire ontology structure; its comparably high value is indicative of the balanced dispersion of concept classes throughout the ontology with consistent, broad representation of the knowledge domain across ontology branches.

**Table 1:** Summary of the structural parameters and their corresponding values measured for PDON

Features	No. of classes	No. of synonyms	Max. depth	Depth variance	Avg. width	Fanout-ness
PDON	631	505	8	1.74	78.8	0.81

### Functional evaluation and gain of knowledge measurement:

The model-based evaluation approach proposed in this work requires that a list of genes and proteins associated to all aspects of PD is being captured by PDON and assessed against the PD disease map as a widely accepted reference (see Evaluation section). Obviously, there is the need to expand the PDON to both coding and non-coding RNA, lipids and eventually to non-coding DNA and modification thereof as well. Since the knowledge space of PD is vast, PDON-driven faceted search enables us to distinguish between the core knowledge directly linked to PD pathophysiology and the emerging novel knowledge surrounding PD pathophysiology (e.g. observations through animal models or epidemiological data). For this purpose, separate queries were performed in the SCAIView environment (accessed on 28.04.2014):

1. a query with all the PDON concepts, which resulted in a list of 16333 human genes/proteins; and
2. PDON branch queries were formulated as ([PDON Node: “<THE BRANCH CONCEPT>”]) AND [MeSH Disease:”Parkinson Disease”] AND [Free Text:” < THE BRANCH NAME>”]

661 human-specific genes/proteins could be extracted from the PD map (i.e. gold standard), which were used for benchmarking the functional performance of the PDON. For this purpose, three branches of the PDON (Etiology, Clinical aspects, Neuropathology) were used to query PubMed abstracts in SCAIView as formulated above (see Methods). Manual curation of the total number of retrieved documents per branch (N_TM_) led to the identification of those genes/proteins that are relevant to the context of the searched branch (TM). Table 2 summarizes these results and shows calculation of the knowledge gain (i.e. the information gained with the support of ontology from text mining in addition to the information already existed in the gold standard, based on the formula described in the Methods section for the knowledge gain calculation) as well as enriched pathways for the gained knowledge to represent the content of this new knowledge. Accordingly, these results demonstrate that PDON-assisted search not only retrieved the majority of proteins already embedded in the PD map gold standard but also captured a large portion of the PD knowledge domain, which has not been represented in the PD disease map so far (gain of novel knowledge from the literature). The rest of genes/proteins that were retrieved by these PDON-driven queries but not found in the PD model represents additional potential knowledge gained from the literature relevant to PD. This new knowledge can be used after expert curation to extend or enrich the current PD disease map and thus, it is important to measure the added value of the potentially novel gained knowledge through the metric that was introduced in the Methods section.

**Table 2:** Parameter values and the final value of the knowledge gain calculated for three major branches of PDON. TM: number of relevant genes/proteins to the branch by PDON; GS: number of genes/proteins extracted from the PD map as gold standard; N: total number of genes/proteins for each branch retrieved by PDON. The queries were performed on Human Genes/Proteins and SCAIView returned lists of unique genes specific to each branch. Numbers represent counts of retrieved genes by SCAIView using PDON

Knowledge domain branches	TM	TM ∩ GS	N_TM_	Gain of novel knowledge	Enriched pathways in the content of new knowledge
Etiology of PD	173	82	273	33 %	MAPK, Chemokine, Adipocytokine, Neurotrophin, Insulin signaling
Clinical aspects of PD	286	97	683	27 %	GPCR signaling, Neuroactive ligand-receptor interactions, Rhodopsin-like receptors, Peptide ligand-binding receptors, Gastrin-CREB signaling
Neuropathology of PD	252	91	471	34 %	Immune system, Signaling by GPCR, Endocytosis, Toll-like receptor signaling, Hemostasis

Table 2 lists parameters of the knowledge gain metric and corresponding novel pathways for selected views of the PDON. The highest percentage of new knowledge by PDON is gained in the branches representing neuropathology, etiology and clinical concepts, respectively. Functional analysis of these additionally identified genes/proteins shows that a couple of statistically significant pathways (in terms of both member proteins and p-values) could be added to the existing PD map.

### Expert evaluation:

PDON-driven information retrieval and extraction can guide analysis of literature in answering complex scientific questions. Experts in the knowledge domain of PD were asked to design complex questions highly relevant for their research work to be posed to the ontology. We selected 2 of these questions that contained concepts specific to the PDON and benchmarked the performance of PDON against PubMed by retrieving literature abstracts that contained hypotheses answering these competency questions. Table 3 provides an overview on the number of total hits as well as the number of relevant documents that were manually verified to contain a hypothetical answer to the corresponding question. Analyses were performed using both SCAIView and PubMed (see Table 3). The following queries were formulated based on the competency questions and were posed to SCAIView and PubMed retrieval systems:

**Table 3:** Results of PDON evaluation based on expert questions. For both competency questions, PDON-driven search in SCAIView retrieved less number of abstracts than simple queries in PubMed but more relevant to the questions (i.e. less noise). This performance efficiency for the PDON-driven search has been calculated in percent as shown in the last column

Competency question number	Total no. of abstracts retrieved by PDON in SCAIView	No. of PDON-derived abstracts answering the questions	Total no. of abstracts retrieved by PubMed	No. of PubMed-derived abstracts answering the questions	PDON-driven retrieval efficiency (% PDON retrieval - % PubMed retrieval)
1	70	20	95	20	28.7 %-21 %: 7.7 %
2	6	5	3	1	83.3 %-33.3 %: 50 %

### Competency question 1. Return all literature references mentioning drugs used to treat 'freezing' in PD.

- Query in SCAIView: [MeSH Disease:"Parkinson Disease"] AND [Parkinson Ontology:"Freezing"] AND [Parkinson Ontology:“Gait disturbance“]) AND [ATC:"ANTI-PARKINSON DRUGS"]) AND [Free Text:“therapy“]
- Query in PubMed: (("parkinson disease"[MeSH Terms] AND gait[Text Word]) AND freezing[Text Word]) AND drug[Text Word])) AND therapy[Text Word]

### Competency question 2. Return literature references containing genes that provide resistance to PD in the animal model MPTP.

- Query in SCAIView: [MeSH Disease:"Parkinson Disease"] AND [Full Text:"resistance"] AND [Parkinson Ontology:"MPTP model"]) AND [Mouse Genes/Proteins]
- Query in PubMed: (((parkinson disease[MeSH Terms]) AND mptp[MeSH Terms]) AND resistance[Text Word]) AND animal model[MeSH Terms]

The PDON-driven semantic search in SCAIView was also able to provide the precise answer to all competency questions by listing drug names (competency question 1), and gene names (competency question 2), as summarized in Table 4. As Medline queries do not return lists of named entities, we could not compare the performance at the entity level.

**Table 4:** Results of PDON-driven search in response to expert competency questions. In contrast to PubMed queries, PDON-driven search in SCAIView generated a list of entities that precisely answer the competency questions

Competency question	Entities	PubMed ID
Return all literature references mentioning drugs used to treat 'freezing' in PD.	Levodopa	6858781, 16222436, 12217618, 15262734
	Selegiline	12112107, 22324564, 18937611
	Amantadine	23185280, 24057149
	Atomoxetine	19361809
	L-threo-DOPS	6337612, 8174332
	Droxidopa	23242741, 7834960
	Manganese	8351000
	Galantamanie	23130517, 18427456
	Methylphenidate	23076544
	Deprenyl	11425939
	Rasagiline	21389939
Return literature references containing genes that provide resistance to PD in the animal model MPTP.	Nos1	12490535, 8643444
	Nos2	10581083
	Sod1	1578260
	Ccl2	17258864
	Mcpt1	17258864

As the results of benchmarking indicate in Table 3 as well, PDON-driven search outperforms PubMed when it comes to specificity (i.e. less noise in terms of the number of retrieved documents) and coverage (i.e. higher number of relevant documents). For less specific queries, PDON performs at the level of PubMed baseline or better. When compared to PubMed, PDON showed higher performance (greater specificity) in retrieving relevant document, i.e. documents containing appropriate answers in the form of hypotheses related to the expert question.

### Application scenario 1: linking etiology view of the PDON to cause-and-effect mechanistic models

One of the biggest scientific challenges we face in the area of NDDs such as AD or PD is the lack of a clear understanding of the alterations at the molecular level that leads to disease manifestation and the very cause of those alterations. As a consequence, the molecular etiology of neither AD nor PD can be described in a formal knowledge representation. In a recent “comment” in Nature Reviews Drug Discovery, Ismael Kola and John Bell called for a new approach towards classifying diseases [19]. They proposed a new taxonomy of disease based on the (presumed) underlying mechanisms. The underlying fundamental notion is that any personalization of treatment requires a deep understanding of the pathophysiological mechanisms and - as a consequence – should be based on a mechanistic understanding of disease etiology.

In the work presented here, we undertake a first attempt at including etiological knowledge in the knowledge representation describing PD. Although this is not yet the “mechanism-based taxonomy” that Kola and Bell called for, it may represent a first step towards integrating mechanistic information on PD – as far as this information can be represented by means of causal and correlative relationships. A dedicated modeling syntax for the representation of causal and correlative relationships is provided by the Biological Language Expression, OpenBEL [20]. OpenBEL is ideally suited to capture knowledge on causal and correlative relationships that may exist at any level of biomedicine. The OpenBEL language can be used for multi-scale and multi-level modeling of any chain of events that may underlie disease.

In the “etiology” section of the PDON, we have encoded those PD-specific disease processes that are comparably well understood in OpenBEL and included these representations directly as “small models” in the PDON. These models can be easily extended, automatically enriched and extracted from the PDON. To exemplify this approach, we have chosen “inherited familial case of PD”, which provides a straightforward scenario for “cause-effect” relationships.

**Fig. 3:**
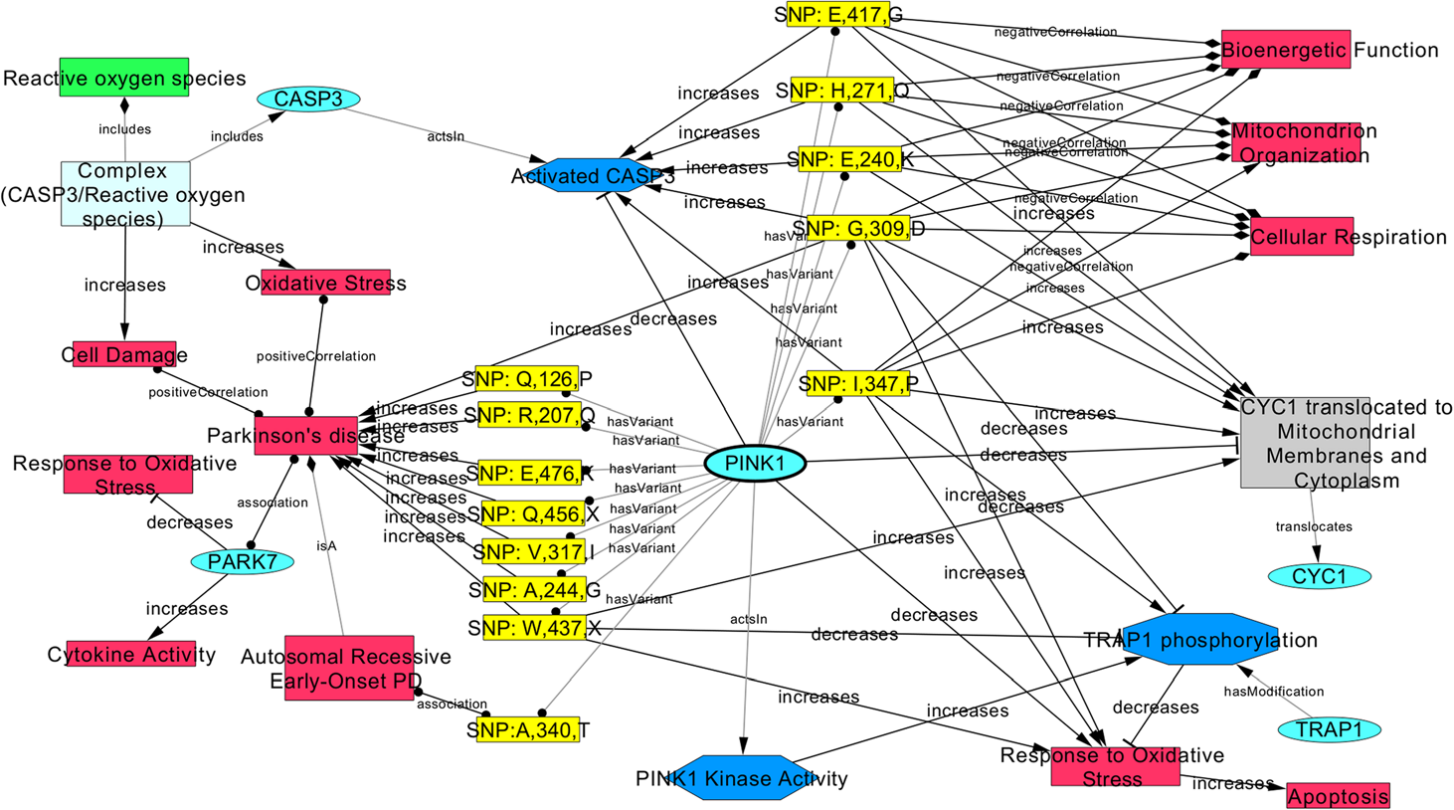
Network visualization of the BEL mechanistic model for causal mutations in PINK1. The model represents the causal association of upstream variants of PINK1 (highlighted in yellow) with downstream pathways and biological processes (highlighted in red). Genes are shown in cyan, intermediary processes in blue, translocation in grey, and reactive oxygen species in green. Relationships have been represented as increase (delta-shaped arrows), decrease (T-shaped arrows), association (diamond-shaped arrows) or variation (circle-shaped arrows). Direct effects have been shown by ‘increase’ or ‘decrease’ annotations on edges whereas indirect effects with unknown intermediate steps are represented by ‘association’ and ‘positive/negative correlation’ relations. Moreover, activation effect of one molecule on another is shown with ‘acts in’, translocation process is annotated with ‘translocates’, and phosphorylation processes have been represented by ‘has_Modification’

A substantial amount of information on the cause (the mutation) and the consequences (at cellular or organ level) is available for parkinsonism. To demonstrate how this information can be transformed into a cause-and-effect model, we used PDON to retrieve mutation information of PINK1. PTEN-induced putative kinase 1 (PINK1) is a mitochondrial serine/threonine-protein kinase encoded by the PINK1 gene and is thought to protect cells from stress-induced mitochondrial dysfunction. Fig. 3 illustrates such a model for pathologic mechanism associated to mutations in PINK1. This model explains how a number of polymorphisms in PINK1 gene antagonize the protective effects of PINK1 by exerting a causal effect on downstream biological processes such as increased response to oxidative stress leading to increased apoptosis, increased molecular activity of CASP3, and negative effect on cellular respiration and bioenergetics in mitochondria. The CASP3 protein is a member of the cysteine-aspartic acid protease (caspase) family and its activation leads to the execution of cell apoptosis. Moreover, the model uncovers the causal effect of PINK1 variants on increased translocation of the CYC1 protein to mitochondrial membranes, which has been shown to result in cell death in neurons [21].

Altogether, this model illustrates the chain of cause-and-effect events (“chain of causation”) starting from the causal effect of defined mutations on cellular processes and ending in clinical manifestations of disease outcomes.

### Application scenario 2: Re-annotation of gene expression data sets with the PDON concept classes

One of the obvious benefits of disease ontologies is their ability to harmonise the annotation of data sets with well-curated terms in their controlled vocabularies. Consistently annotated gene expression data sets will facilitate the automated identification of comparable studies in systematic meta-analyses that aim to identify patterns of expression or interesting relationships between expressed genes that may come to light in the context of multiple studies. Gene expression studies can be found in the GEO (Gene Expression Omnibus) database, which is publicly available [22]. To demonstrate the use of PDON for the re-annotation of gene expression data sets we have selected from the GEO database two examples for gene expression data sets with rather heterogeneous annotations:

GSE 16658 ; as an example of a data set with rather limited annotation

GSE 32037 ; as an example of a GEO data set with comparably rich annotation

**Fig. 4:**
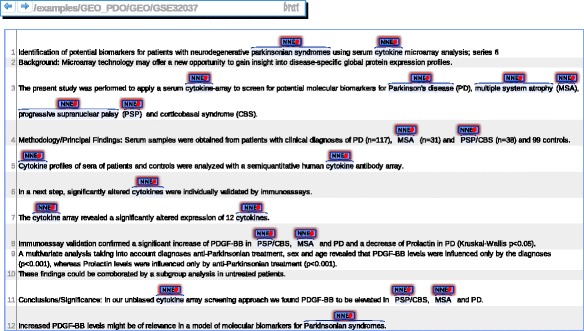
Rich annotation of the description field for the GSE 32037 entry in the GEO database using PDON

**Fig. 5:**
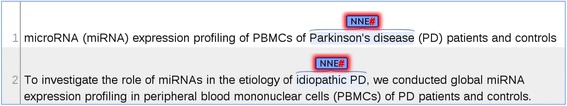
Limited annotation information in a relevant gene expression data set. The description of GSE 16658 states the purpose of the study and provides some information on the type of cells (PBMCs) used for the isolation of patient samples

The rich annotation of GSE 32037 becomes obvious, when the “description field” of this GEO entry is subjected to a text-mining pipeline that identifies PDON terms in text (Fig. 4). In contrast, the description field of the GSE 16658 data set is rather limited with respect to information content (Fig. 5).

The observed lack of detail in the description of gene expression data fields is a common phenomenon with many primary data repositories; ArrayExpress – a database for functional genomics experiments including gene expression studies [23]- therefore is systematically re-annotating all data sets, but that is a manual process done by expert curators who usually follow annotation guidelines that are valid for a broad spectrum of indication areas and thus do not represent specific information at great detail.

More detailed information about the cohort of patients is, however, often available from the primary publication describing the data set. In our example, the respective data set has been described in a publication that is available as open access. The terms and conditions of the journal do allow automated analysis of the full text. Therefore, the complete full text publication describing GSE 16658 was subjected to automated analysis with PDON terms [24].

The complete full text publication describing GSE 16658 was subjected to automated analysis with terms from the PDON terminology using a UIMA-based annotation workflow. Unstructured Information Management Architecture (UIMA) is content analytics software, which is used for natural language processing in large sets of unstructured text such as biomedical publications [25]. Detailed information on the patient cohort underlying the study is tagged and indicated by mark-ups (Additional file 1). Moreover, the ubiquitin pathway, which has been identified as a pathogenic pathway in this publication, was highlighted by PDON repeatedly. As demonstrated in this example, curation teams can readily make use of automatically pre-annotated text to enrich the annotation of GSE 16658 with the PDON terminology.

## Discussion

Disease-specific ontologies are emerging as powerful semantic tools, which go beyond the scope of GO and offer high semantic specificity and sensitivity by placing the disease context at the center. This trend is indicative of the fact that successful development of novel biomarkers and drugs requires an integrated systems view of the pathophysiological mechanisms that underlie disease, which is highly geared towards understanding connections across multiple biological scales, from molecular interactions to disease phenotypes. Among chronic and complex diseases, NDDs are the most challenging diseases to deal with and present a high level of heterogeneity ranging from diverse molecular mechanisms, various cell types and anatomical regions to different levels of clinical manifestation. Thus, harmonization of such high diversity at the data level and enormous heterogeneity at the clinical level needs high-resolution representation of the knowledge domains specific to NDDs.

In the field of NDDs disease-specific ontologies such as epilepsy ontology [26] and Alzheimer’s disease ontology [17] have been recently introduced. However, most potential users of such ontologies including molecular biologists and clinicians find the notations of such formal representations counter-intuitive and non-reusable. PDON not only provides a standard terminology and platform for interoperability of the PD knowledge domain among research groups, but also supports users via literature search through its lexicalized form. The broad coverage of PD-specific concepts at various granularity levels ensures that PDON enables users to capture and integrate PD-related information for systems analysis purposes. In terms of specificity of the PDON search, we found that the maximum overlap of the PDON-derived gene list with the PD map-derived genes is reached in each query within the top 50 genes returned by SCAIView and the most interesting genes missing in the PD model were then identified after this rank position. The PDON search covered more than 90 % of the gold standard entity list from the PD map reference when the entire knowledge domain of PD was queried, which shows the high sensitivity of the PDON. However, the low gene overlap between the gold standard (the reference PD map) and the PDON-derived list of genes (Table 2) is indicative of the large amount of peripheral information that has been used in the construction of the PD disease map complementary to the core pathology. For instance, among the novel knowledge gained by PDON is ATP5I protein, which is a part of the ATP synthase complex in the mitochondrial ROS cascade and complements the core cascades involved in mitochondrial dysfunction under PD condition. In the reference PD map, however, this protein has not been shown and only complex V has been presented. On the other hand, this comparison suggests that PDON-derived results contain rich information content relevant to the core pathophysiology of PD, which is still absent in the PD disease map and can be potentially included in the next version. Introduction of the gain of knowledge measurement metric made it possible to quantitatively measure the gain of novel information content captured by PDON. Moreover, representation of the gained knowledge by enriched pathways confirms the novelty of the gained knowledge in comparison to the reference PD map, as these pathways are not present in the current reference PD map.

An important feature of the PDON is its capacity to project multiple perspectives of the PD knowledge domain among stakeholders in both academia and industry from molecular biology to epidemiological and clinical studies. For instance, during the construction and separate rounds of curation with both clinicians and molecular biologists, special attention was paid to bridging the gap between two higher-level perspectives, namely the perspective of clinicians and the perspective of drug developers. Due to the representation of different perspectives in the domain of PD research, we do hope that the PDON will be broadly adopted and used for the exchange of data, annotation of existing data sets and the communication of knowledge.

The unique value that application of PDON can provide to the PD research community was demonstrated by using PDON for the rapid construction of mechanistic BEL models. This ability can be used for representation as well as visualization of the PD knowledge subdomains in the form of cause-and-effect systems models. As demonstrated in the application scenario, mutations in the PINK1 gene is well known for their causal association to familial early onset PD but their mechanistic model reveals their potential involvement in the etiology of sporadic late onset PD as well. This feature of the PDON, therefore, can be used for investigating associations between familial and sporadic PD in general, and associations between parkinsonism syndromes in particular at the mechanism level. To this end, the authors foresee that one potential application of PDON could be revision of the current classification of PD syndromes based on etiological mechanism underlying each syndrome.

## Conclusions

In the times of re-orientating PD research towards systems modeling and analysis, the Parkinson’s disease ontology serves as a semantic framework for standardization and harmonization of a large amount of heterogeneous data and knowledge in the field of PD. Indeed, PDON delivers the knowledge domain of PD in a compact, computer-readable form, which can be further used to construct, represent and automatically extend PD-related computable models. Beside advantages, PDON has its own limitations such as missing concepts, lack of standard definitions, or incompleteness of synonym list. Addressing these shortcomings requires constant contribution of the PD research community to the betterment of the current version of PDON.

## Methods

The PDON has been constructed in accordance to the ontology-building life cycle [27]. Such a methodology – compared to other methods - offers a set of defined stages when building ontologies to assist identifying construction principles for each stage as well as relationships among stages.

### Knowledge acquisition and conceptualization

A first collection of terms and concepts related to PD was generated by scanning various knowledge sources. Initially we used a list of sources recommended by Parkinson’s experts (i.e. neuroscientists and clinicians) including medical text books such as Parkinson’s Disease and Movement Disorders [28] and encyclopedias like Encyclopedia of Parkinson’s Disease [29]. After extracting the key concepts manually, we then used the search functionality of Google (such as Books and Scholar) to find online resources that contain additional concepts describing the knowledge domain of PD (e.g. www.parkinsons.org). Other resources including review articles and content of online books were used in this way and any available hierarchical organization (structure) of the concepts was extracted along with the concepts themselves. Corresponding definitions and references were also included. Concept enrichment was assisted by n-gram analysis so that publication abstracts were scanned for 2-grams to 5-grams, describing meaningful terms with 2 to 5 words, by using a java program written for this purpose. These n-grams underwent manual inspection and relevant terms were added to the ontology.

### Formal representation

The Protégé OWL editor was used as a tool for building the PDON in Web Ontology Language (OWL) format [30]. Concept classes were further annotated with synonyms.

### Structural evaluation

Structural features of the ontology were computed using an existing java script [31]. These features include topological and logic properties such as: depth and breadth (related to the cardinality of paths in a graph), tangledness (related to multi-hierarchical nodes), and fan-outness (related to the dispersion of nodes).

### Functional evaluation

Functional performance of the ontology was measured through a novel model-based evaluation approach. In this approach, the assumption is that the expert-curated molecular map of PD, which has been recently published by Fujita et al. (2013), represents a good part of the current knowledge about molecular processes related to PD and is considered as the gold standard [32]. A list of genes and proteins in this map was used for benchmarking the list of genes and proteins that had been retrieved from the literature through PDON-supported literature mining.

### Expert evaluation

The expert panel’s revision of the ontological structure is considered as a genuine evaluation for disease ontologies [33]. Revisions of the PDON drafts were initially performed in the presence of an expert panel of 6 experts in PD from UCB Pharma, composed of molecular biologists, clinicians and physicians, who generated the “pharma view” of the ontology. In order to generate the “clinical view” of the ontology, the PDON underwent a manual curation by clinician experts in the field of PD (UW and DS).

### Ontology-driven information retrieval and extraction

Transformation of the ontology OWL format into a dictionary file was achieved using a Java program that extracts the concept names and the corresponding synonyms from the ontology OWL structure and assigns unique identifiers to each concept which can be stored in form of a dictionary. This dictionary was incorporated into the text-mining tool ProMiner [34] and results were deployed in the semantic search engine SCAIView for context-sensitive visualization of query results. SCAIView is a semantic search engine that provides a text mining-based environment for information retrieval and extraction from PubMed publications using various terminologies and ontologies [35]. SCAIView environment can be freely accessed under http://www.scaiview.com/scaiview-academia.html; PubMed portal can be accessed through http://www.ncbi.nlm.nih.gov/pubmed.

### Novelty analysis

Current disease models often suffer from the limited information content and are usually representative of the well-established knowledge at the core of disease knowledge domain. Application of ontology-driven information retrieval technology helps to extend the core knowledge domain to its boundaries by automated capturing of novel biological entities (here: genes/proteins) that represent the less established or emerging knowledge (gain of novel knowledge). We introduce a metric for the measurement of novel complementary knowledge using the following formula:

$$ \mathrm{Novelty}\left({\mathrm{L}}_{\mathrm{TM}}\vert {\mathrm{L}}_{\mathrm{GS}}\right)=\left(\left|\mathrm{T}\mathrm{M}\right| - \left|\mathrm{T}\mathrm{M}\ \cap\ \mathrm{G}\mathrm{S}\right|\right)\ /{\mathrm{N}}_{\mathrm{TM}} $$

Where:

- L_TM_ = the “curated” result list from text-mining (TM) algorithm
- L_GS_ = the result list from the reference gold standard
- N_TM_ = the total number of entities retrieved from TM
- TM: Relevant (true positive) entities (i.e. genes/proteins)
- GS: Gold standard list of entities
- TM ∩ GS: Overlap between entities retrieved by TM and entities extracted from GS

To show the biological content of the novel knowledge gain by this approach, the list of novel genes/proteins retrieved by PDON (i.e. |TM| - |TM ∩ GS|) was subjected to the pathway enrichment analysis using MsigDB [36].

### Mechanistic model building

Around PINK1 gene, which is involved in the pathology of familial PD, a “pathophysiology mechanism” model was built based on OpenBEL, a modeling language ideally suited to represent causal and correlative relationships. PDON was used in the SCAIView environment to formulate the following query for PINK1 mutations:

- (([SNPs]) AND [Human Genes/Proteins:”PINK1”]) AND [PDON]

**Fig. 6:**
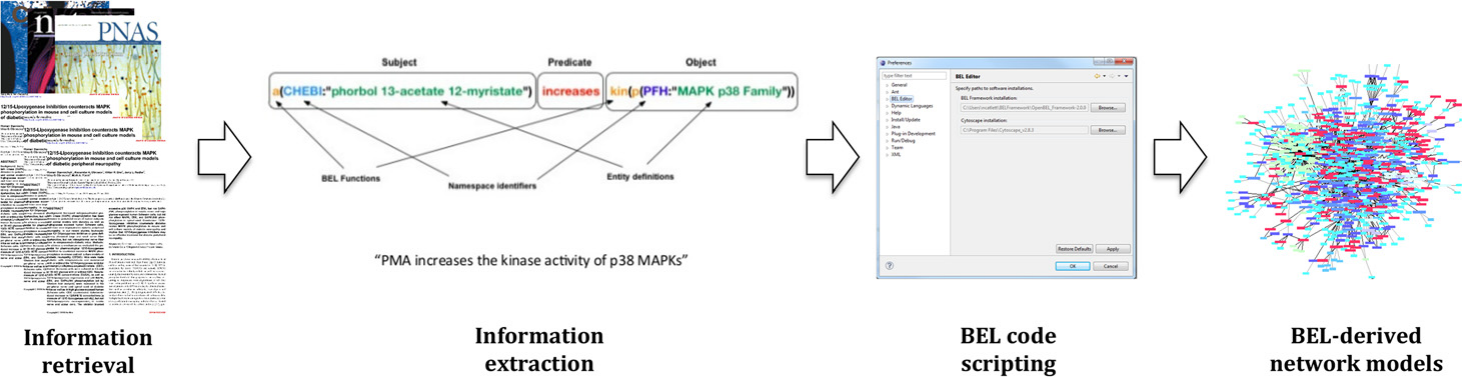
Overview of the overall workflow used for construction of mechanistic BEL models

Retrieved abstracts were checked manually for the relevance of their information content and then subjected to the process of BEL coding by manual extraction of the < subject – relationship – object > predicates and converting them into the BEL script in the BEL-editor software [37]. These scripts were converted into network models with functionality of the BEL-editor and visualized by the Cytoscape software [38]. The overall workflow of mechanistic model building has been illustrated in Fig. 6.

## Additional file

Additional file 1:
**Convergence of miRNA Expression Profiling, ?-­Synuclein Interacton and GWAS in Parkinson's Disease.** (DOCX 449 KB)
