# Supplementary material for: PDON: Parkinson’s disease ontology for representation and modeling of the Parkinson’s disease knowledge domain
Source: Theor Biol Med Model. 2015 Sep 22;12:20. doi: 10.1186/s12976-015-0017-y (PMC4580356; doi:10.1186/s12976-015-0017-y)
Supplement: Additional file 1: — Convergence of miRNA Expression Profiling, ?-­Synuclein Interacton and GWAS in Parkinson's Disease. (DOCX 449 KB) [file 12976_2015_17_MOESM1_ESM.pdf]

/examples/GEO_PDO/PMC/22003392 brat

1 Convergence of miRNA Expression Profiling, ?-Synuclein Interacton and GWAS in Parkinson's Disease.

2 ABSTRACT.

3 miRNAs were recently implicated in the pathogenesis of numerous diseases, including neurological disorders such as Parkinson's disease (PD).

4 miRNAs are abundant in the nervous system, essential for efficient brain function and play important roles in neuronal patterning and cell specification.

5 To further investigate their involvement in the etiology of PD, we conducted miRNA expression profiling in peripheral blood mononuclear cells (PBMCs) of 19 patients and 13 controls using microarrays.

6 We found 18 miRNAs differentially expressed, and pathway analysis of 662 predicted target genes of 11 of these miRNAs revealed an over-representation in pathways previously linked to PD as well as novel pathways.

7 To narrow down the genes for further investigations, we undertook a parallel approach using chromatin immunoprecipitation-sequencing (ChIP-seq) analysis to uncover genome-wide interactions of ?-synuclein, a molecule with a central role in both monogenic and idiopathic PD.

8 Convergence of ChIP-seq and miRNomics data highlighted the glycosphingolipid biosynthesis and the ubiquitin proteasome system as key players in PD.

9 We then tested the association of target genes belonging to these pathways with PD risk, and identified nine SNPs in USP37 consistently associated with PD susceptibility in three genome-wide association studies (GWAS) datasets (0.46?OR?0.63) and highly significant in the meta-dataset (3.36?10?4<p<1.94?10?3).

10 A SNP in ST8SIA4 was also highly associated with PD (p?=?6.15?10?3) in the meta-dataset.

11 These findings suggest that several miRNAs may act as regulators of both known and novel biological processes leading to idiopathic PD.

12 INTRODUCTION.

13 Parkinson's disease is a neurodegenerative disorder clinically characterized by bradykinesia, muscle rigidity, resting tremor and, in more advanced stages, postural instability.

14 Its main pathological hallmark is the loss of over 70% of the dopaminergic neurons (DNs) within the substantia nigra (SN), leading to functional deficits in the basal ganglia circuitry due to reduced levels of dopamine.

15 The identification of several genes causing monogenic forms of PD (e.g. ?-synuclein [SNCA], Parkin, DJ-1, PINK1, LRRK2) has led to the generalized view that protein misfolding, mitochondrial dysfunction, impaired oxidative stress response, and altered function of the ubiquitin-proteasome system are central pathogenic mechanisms underlying the familial forms of PD [1].

16 However, far less is known about the molecular mechanisms underlying idiopathic PD.

17 miRNAs have recently emerged as an important class of small RNAs (?22 nucleotides) that act as post-transcriptional regulators of gene expression by base-pairing with their target mRNAs.

18 They regulate neuronal processes such as brain morphogenesis, neuronal cell fate and differentiation, and transcription of neuronal-specific genes [2].

19 Recent studies have linked several miRNAs to sporadic PD.

20 miR-133b was found to be specifically enriched in midbrain DNs of normal individuals and reduced in PD patients [3].

21 In vitro, miR-133b was found to regulate DN maturation and function through a negative feedback loop with Pitx3, a transcription factor that activates midbrain DN gene expression [3].

22 miR-433 binds to a polymorphism in the promoter region of the fibroblast growth factor 20 gene (FGF20) which is associated with PD [4].

23 The risk allele disrupts the binding site of miR-433, resulting in enhanced translation of FGF20 and increased ?-synuclein expression [4].

miR-7 and miR-153 were shown to be predominantly expressed in the brain and to regulate ?-synuclein expression levels [5]?[6].

25 In addition, mutant LRRK2 was found to negatively regulate translational repression mediated by miRNAs [7].

26 None of these studies reported genome-wide miRNA expression profiling in the blood of PD patients.

27 Since previous studies were restricted to brain tissue and limited in the number of samples and miRNAs evaluated, we sought to investigate the role of miRNAs in idiopathic PD by performing microarray expression profiling in blood samples of PD patients and controls.

28 ?-synuclein is a key player in PD.

29 It is the main component of the protein aggregates accumulated in the brain of patients, a pathologic hallmark of this disease.

30 Further support for the implication of this molecule in PD has been gathered from GWAS [8]?[13] where the genetic architecture of the most common forms of PD has been investigated.

31 Genome-wide significance has been reached in three of these GWAS [11]?[13], and SNCA and the MAPT region have been confirmed as major PD risk loci.

32 In addition, mutations (e.g. A53T, A30P, E46K, and gene duplications and triplications) and polymorphisms in SNCA are linked both to monogenic and complex forms of PD, respectively [14].

33 Despite its recognized importance, the molecular mechanisms underpinning its role in PD are not yet clear.

34 Although pathways like the ubiquitin proteasome system and chaperone-mediated autophagy have been implicated in ?-synuclein turnover, our knowledge of its biology is still limited [15].

35 Here, we used ChIP-seq as an unbiased approach to identify ?-synuclein binding sites genome-wide.

36 This technology captures the full spectrum of actions of this molecule across the genome, providing information that will not only provide insights into the putative roles of ?-synuclein in the nucleus, but also connect it to previously unanticipated cellular processes.

37 miRNAs have been studied for their direct or indirect action on ?-synuclein [4]?[6] but they are predicted to target a wide range of genes.

38 Given the centrality of ?-synuclein in PD, here we propose to go one step further and not only to look at its interactions with other molecules and pathways but also to look into the cross-talk between the ?-synuclein interactome and miRNAs.

39 To identify novel genes and pathways associated with PD, we followed an integrative approach, intersecting the results from these complementary genome-wide analyses to further test them in a GWAS meta-dataset, gaining further insight into the molecular mechanisms involved.

40 Importantly, a PD miRNA signature with 18 miRNAs was obtained and two pathways (the glycosphingolipid biosynthesis - ganglioseries and the protein ubiquitination pathway) were highlighted as key players in PD pathogenesis.

[illegible]

41 Furthermore, 3 miRNAs emerged as main modulators of these two pathways (miR-30b, miR-30c and miR-26a), and two genes (USP37 and ST8SIA4) were found significantly associated with PD susceptibility.

42 RESULTS.

43 MIRNA EXPRESSION PROFILING.

44 The principal demographic and clinical characteristics of the nineteen unrelated idiopathic PD patients and thirteen controls included in the miRNA profiling study are summarized in Table 1.

45 Recruited patients covered the full spectrum of early to advanced PD (Hoehn & Yahr stage 1 to 5) and age was comparable across the two groups (average age-at-examination ? SD of 65.1?4.4 years in patients and 64.4?5.9 years in controls).

46 We conducted miRNA expression profiling in PBMCs of the study participants using Exiqon-developed miRCURY?

47 LNA microarrays spotted with four replicate probes for 763 human miRNAs (733 from miRBase (http://www.mirbase.org/) database release 13 plus 30 miRPlus novel human miRNAs from Exiqon).

48 We found that 490 of these miRNAs were expressed in our samples.

49 Differentially expressed miRNAs were identified using a linear modelling approach for each miRNA to estimate the fold-changes and standard errors, followed by an empirical Bayes smoothing to moderate the standard errors [16].

50 Empirical Bayesian methods provide more stable results even when the number of arrays is small.

51 Taking a B-statistic (logarithm of the posterior odds that a gene is differentially expressed) threshold of 1, eighteen miRNAs were found differentially expressed (Table 2).

52 Interestingly, all of these miRNAs are under-expressed in PD samples (fold-changes ranging from ?1.41 to ?3.02).

53 To globally validate the microarray results, we performed qRT-PCR for five miRNAs, three of which had negative fold-changes (miR-126*, miR-32, and miR-101), and two had positive fold-changes (miR-15b and miR-550) in PD cases.

54 Only miR-126* was significantly under-expressed in our microarray experiment (B?1), while the other four miRNAs tested had B-statistics below 1.

55 qRT-PCR supported our microarray findings (Figure S1) in terms of directionality (positive versus negative fold-changes) and strength of significance of differential expression (the lowest p-value in the qRT-PCR experiment was obtained for miR-126* which was the only miRNA among those five with a B-statistic greater than 1).

56 Unsupervised hierarchical clustering (HC) of the samples using the 18 differentially expressed miRNAs reveals that PD cases and controls cluster into separate groups with the exception of controls C2, C3, C7, and C13 and cases P18 and P19 (Figure 1).

57 A similar pattern is observed using the principal component analysis (PCA) (Figure S2), where the first principal component explains 84.1% of the variance and allows a clear separation of most samples according to affection status.

58 Supporting the consistency of our results, analysis of variance revealed that the main source of variation in the expression data is the individual's affection status (Figure S3), and we typically observed similar expression patterns for miRNAs derived from the same stem-loop precursor (e.g. co-clustering in Figure 1 of miR-199a-5p and miR-199a-3p, and of miR-151-5p and miR-151-3p, which derive from the miR-199a and miR-151 stem-loop precursors, respectively).

59 PREDICTED TARGET GENES AND PATHWAY ANALYSIS.

60 In an effort to place these miRNA expression profiling findings into a biological context and to generate new testable hypotheses, and given that miRNAs have very few experimentally validated targets, we first performed an in silico search for predicted target genes of the 18 differentially expressed miRNAs.

61 In view of the fact that no particular target prediction software has consistently shown to be superior to all others, we used the ?Predicted Targets?

62 tool in the miRecords resource [17] which integrates the results of eleven established miRNA target prediction algorithms.

63 Six softwares (miRanda v.1.9 [18], MirTarget2 v.2.0 [19], PicTar [20], PITA v.6 [21], RNAhybrid v.2.2 [22], and TargetScan v.4.1 [23]) generated almost all (>95%) of the predicted target genes for the miRNAs of interest.

64 These six softwares rely on very distinct principles and methods, namely near-perfect base complementarity in the ?seed?

65 region, evolutionary interspecies conservation, miRNA-mRNA duplex thermodynamic stability, target site accessibility, and machine learning techniques.

66 Given that these predictions have not been experimentally validated, we opted for a stringent threshold of at least six softwares to select the predicted miRNA target genes for follow-up.

67 In this case, seven miRNAs (miR-374a, miR-199a-3p/miR-199b-3p, miR-126*, miR-151-5p, miR-126, miR-147, and miR-374b) had no predicted target genes, and the remaining eleven miRNAs were predicted to target 662 unique genes (Table S1).

68 To correlate expression findings with relevant biological processes and pathways, we performed pathway analysis using the manually-curated literature-based Ingenuity Pathway Analysis (IPA) database.

69 Among the 662 unique predicted target genes used to query the database, 553 were eligible for functions/pathway/list analyses.

70 The top ten canonical pathways over-represented among our target genes were (Figure S4A and Table S2): glycosphingolipid biosynthesis - neolactoseries (5 molecules), semaphorin signaling in neurons (7 molecules), DNA methylation and transcriptional repression signaling (4 molecules), retinoic acid receptor (RAR) activation (13 molecules), hypoxia signaling in the cardiovascular system (8 molecules), glycosphingolipid biosynthesis - ganglioseries (4 molecules), O-glycan biosynthesis (4 molecules), protein ubiquination pathway (16 molecules), synaptic long-term potentiation (9 molecules), and nicotinate and nicotinamide metabolism (8 molecules).

71 ?-SYNUCLEIN TARGET GENES.

72 High-throughput microarray studies typically generate too many leads to follow-up, requiring subsequent validation steps.

73 To narrow down the genes for further investigation in association studies, we intersected the results from the miRNA pathway analysis with those from a study aimed at unveiling genomic targets for ?-synuclein modulation (interacton), which may suggest novel unanticipated pathogenic pathways in PD.

74 To identify ?-synuclein binding sites throughout the genome, we performed chromatin immunoprecipitation (ChIP) with an anti-?-synuclein antibody in cultured human H4 neuroglioma cells expressing endogenous levels of the protein.

75 Next, high-throughput sequencing of the co-immunoprecipitated genomic DNA fragments was performed.

76 Mapping the sequenced fragments to the reference genome gives a profile of the DNA regions that are enriched (?peaks?) in the pulldown, with a peak being defined as a region that contains significantly more reads from an experimental pulldown than from a negative control (ChIP sample and total input, respectively).

77 The analysis revealed 7666 statistically significant peaks corresponding to 238 unique genes, ranging in size from 100?416 bp (Table S3).

78 The quality of the chromatin and specificity of the antibody were confirmed by ChIP analysis using the CDC-42 promoter, previously reported as a putative target of ?-synuclein.

79 Additionally, the promoter enrichment was assayed using the NEDD4 promoter sequence, shown in our analysis to significantly bind ?-synuclein (NEDD4 belongs to the NNE# ubiquitin proteasome pathway).

80 Real-time PCR analysis of the immunoprecipitated DNA resulted in a significant enrichment for ?-synuclein binding to both the CDC-42 and NEDD4 promoter, when compared to the myoglobin exon 2 non-binding control region (2.36- and 1.51-fold enrichment for CDC-42 and NEDD4, respectively).

81 IPA analysis revealed that the top ten canonical pathways over-represented among the 238 genes targeted by ?-synuclein were (Figure S4B): fatty acid biosynthesis (ACACB, OLAH), mechanisms of viral exit from host cells (NEDD4, PRKCG, VPS24), aldosterone signaling in epithelial cells (ACCN1, NEDD4, PIP5K1C, PRKCG), PPAR signaling (IL1R2, INSR, MAP4K4, PDGFB), hepatic cholestasis (ESR1, GCGR, IL1R2, INSR, PRKCG), protein ubiquitination pathway (AMFR, NEDD4, PSMD1, UBR2, USP3, USP6), rac signaling (ANK1, MCF2L, PAK3, PIP5K1C), clathrin-mediated endocytosis signaling (AMPH, FGF12, FGF13, PDGFB, PIP5K1C), glycosphingolipid biosynthesis - ganglioseries (ELOVL2, ST8SIA1), and ephrin receptor signaling (EPHA6, MAP4K4, PAK3, PDGFB, WIPF1).

82 We hypothesized that ?-synuclein ChIP peaks/pathways common to predicted target genes/pathways from our miRNA analysis, particularly in the top ten pathways, would highlight important genes and pathways implicated in PD pathology.

83 Glycosphingolipid biosynthesis - ganglioseries together with the protein ubiquitination pathway emerged in both the miRNome and the interacton IPA analyses, with three common genes, USP6, NEDD4, and USP3, in the protein ubiquitination pathway.

84 These three genes are also putative targets of three miRNAs (miR-30b, miR-30c, miR-26a) over-represented in the miRNomic pathway analysis (Table S2).

85 ASSOCIATION STUDY.

[illegible]

86 Given that the convergence of the miRNomics and the ?-synuclein interacton approaches highlighted multiple genes in the glycosphingolipid biosynthesis and the protein ubiquitination pathways previously been linked to PD [24]?[27], we further investigated the association of the genes in these pathways with risk for PD.

87 Six independent genome-wide association studies investigated the genomic susceptibility to PD [8]?[13], and we performed a joint analysis of the three datasets to which we had access [9], [10], [13].

88 The meta-dataset in this study includes 1752 PD cases and 1745 controls.

89 We tested the association of 388 SNPs in 20 genes predicted to be targets of 11 out of the 18 differentially expressed miRNAs belonging to the glycosphingolipid biosynthesis - ganglioseries and the protein ubiquitination pathways (Table S4).

90 The number of polymorphisms tested in each gene and the markers with positive association findings are summarized in Table 3.

91 USP37 (ubiquitin specific peptidase 37) in the protein ubiquitination pathway showed the strongest associations with nine SNPs having p-values between 3.36?10?4 and 1.94?10?3 (Table 3).

92 All nine SNPs are in high linkage disequilibrium with each other (r2>0.9).

93 The most significant SNP rs13027934 (p?=?3.36?10?4 in the meta-analysis) displayed a consistent association trend in each GWAS dataset: in the Hussman Institute for Human Genomics (HIHG) dataset (OR?=?0.57, p?=?0.13), in the National Institute of Neurological Disorders and Stroke (NINDS) dataset (OR?=?0.46, p?=?0.02), in the Center for Inherited Disease Research (CIDR) dataset (OR?=?0.55, p?=?0.02).

94 Consistent evidence for association was also found for rs2059198 in ST8SIA4 (ST8 alpha-N-acetyl-neuraminide alpha-2,8-sialyltransferase 4) in the glycosphingolipid biosynthesis pathway (OR?=?1.20 and p?=?6.15?10?3 in the combined GWAS, OR?=?1.22 and p?=?0.08 in the HIHG dataset, OR?=?1.17 and p?=?0.36 in the NINDS dataset, and OR?=?1.19 and p?=?0.05 in the CIDR dataset) (Table 3).

95 The effect sizes of those SNPs are small, which is expected for complex diseases: the median odds ratio for associated SNPs in GWAS is 1.3 [28].

96 As a result, none of these SNPs would survive the stringent Bonferroni correction for multiple testing given the sample size.

97 The trend for association (in terms of p-values and ORs) in several GWAS datasets and in the meta-analysis, however, supports the possible involvement of these genes in PD etiology.

98 In addition, there is evidence of association with PD in the meta-analysis for SNPs rs7315790 (p?=?3.55?10?2) and rs10784293 (p?=?2.26?10?2) in USP15 (ubiquitin specific peptidase 15), rs10450989 (p?=?3.97?10?2) in USP3 (ubiquitin specific peptidase 3), and rs878396 (p?=?1.04?10?2), rs4149589 (p?=?1.96?10?2), and rs2043265 (p?=?2.49?10?2) in NEDD4L (neural precursor cell expressed, developmentally down-regulated 4-like), further supporting the possible involvement of these pathways in PD etiology.

99 DISCUSSION.

100 Our data identified a signature of eighteen miRNAs that permits a good separation among PD cases and controls.

101 All of these 18 miRNAs are under-expressed in patients.

102 The slight down-regulation of these miRNAs is predicted to lead to a mild up-regulation of their target genes.

103 A modest over-expression of susceptibility genes may, over time, lead to a late-onset neurodegenerative disease such as PD [29]?[30].

104 The miR-133b, miR-433 and miR-153 miRNAs previously implicated in PD [3]?[6] were not expressed at detectable levels in the PBMCs samples assayed here, and therefore their role in PD could not be assessed in this study.

105 On the other hand, miR-7 was expressed, but not deregulated in PD.

106 Similar results for miR-133b (not expressed) and miR-433 (not deregulated in PD) were obtained on laser microdissected postmortem dopaminergic neurons in a PD case-control study [29].

107 Discrepancies in miRNA expression results across studies may result from technical (e.g. sample preparation) as well as biological (e.g. tissue) differences.

108 In our study, we chose to focus on PBMCs not only due to the obvious practical advantages and potential clinical applications of these cells, but also due to important biological considerations.

109 Over 70% of the substantia nigra of PD patients is composed of dead or dying cells at the time of diagnosis, and therefore expression studies in SN tissue of PD patients is likely to reveal downstream unspecific processes (such as programmed cell death and survival) rather than pointing to the etiopathogenic mechanisms.

110 Furthermore, brain-enriched miRNAs have limited stability and relatively short half-lives (?1?3.5 h) in human brain tissues, even with very short post-mortem brain freezing intervals (?1 h) [31].

111 There is communication between the brain and immune system through multiple mechanisms that may be reflected in blood cells [32], and PBMCs share more than 80% of the transcriptome with other tissues types including brain [33].

112 Finally, previous expression profiling studies for neurodegenerative diseases in PBMCs have uncovered important and novel findings [34], and the miRNA expression pattern in normal brain appears to be more similar to PBMCs than to other tissues [35].

113 The mis-classification of several individuals in HC and PCA analyses could not be correlated to any experimental artefact or error (such as collection date or sample switching), nor to any study participant characteristic (such as age, sex, medication).

114 Even though mis-clustering of individuals is characteristic of high-throughput methods [36], it might have been resolved by the inclusion in the expression study of samples from patients with other parkinsonian syndromes with involvement of the SN such as progressive supranuclear palsy, frontotemporal dementia with parkinsonism [37], and corticobasal degeneration.Consistent with the view that idiopathic PD most likely requires multiple insults in different biological processes before neuronal loss occurs [38], our ?miRNA-derived IPA analysis?

115 revealed that several of the top canonical pathways over-represented among our target genes have been previously linked to PD.

116 This is the case for pathways such as the semaphorin signaling in neurons and the DNA methylation and transcriptional repression signaling.

117 Semaphorins are axon guidance proteins playing important roles in the mesencephalic dopamine neuron system development during embryogenesis.

118 A GWAS showed that a SNP in SEMA5A was the most significantly associated with PD [8].

119 However, four subsequent case-control replication studies reported contradictory results [39]?[42].

120 DNA methylation and transcriptional repression signalling was the third most significantly over-represented pathway.

121 Mechanisms regulating gene expression, whether through DNA methylation or signalling pathways such as checkpoint transduction cascades or transcriptional repression, are very likely associated to PD and regulated by miRNAs.

122 Saijo et al. demonstrated that Nurr1/CoREST transrepression pathway attenuates neurotoxic inflammation, protecting against loss of dopaminergic neurons in PD [43].

123 Also, Zhong et al. presented DJ-1 as a potential regulator of protein sumoylation and directly link the loss of DJ-1 expression and transcriptional dysfunction to impaired dopamine synthesis [44].

124 Genetic and biochemical studies have established a central role for ?-synuclein accumulation in the pathogenesis of Parkinson disease.

125 In this study, we identified a large set of putative ?-synuclein target (interacting) genes in human H4 neuroglioma cells, which we extensively use as a model for studying the molecular basis of PD [45]?[46], providing the first insight into the interacton of endogenous ?-synuclein.

126 Pathway analysis of this interacton suggested several primary targets of ?-synuclein, with the glycosphingolipid biosynthesis and the protein ubiquitination pathways being common to the miRNome IPA analysis.

127 Data mining of these pathways in three GWAS studies highlighted the consistent associations of USP37 and ST8SIA4 with PD and gave further support to the involvement of glycosphingolipids and the ubiquitin proteasome system in the physiopathology of PD.

128 Furthermore, 3 miRNAs (miR-30b, miR-30c and miR-26a) which are among the most abundant miRNAs in primary human neuronal and glial cells [31] and simultaneously involved in the regulation of ?-synuclein interacting genes, emerged as the main modulators of these two pathways in our expression analyses.Glycosphingolipids and their sialic acid-containing derivatives, gangliosides, are important cellular components and abundant in the nervous system.

129 They are known to undergo dramatic changes during brain development, but our knowledge on the mechanisms underlying their quantitative and qualitative changes is still fragmentary [47].

130 Glycosphingolipids are closely related to the ceramide metabolism that has already been linked to PD through the glucocerebrosidase (GBA) gene [26]?[27].

131 In addition to being the major non-lysosymal system for degrading proteins in the cell, the ubiquitin proteasome system (UPS) regulates function and translocation of proteins, many of which play a role in the determination of cell fate.

132 Protein mediators of apoptosis are regulated by the UPS, via direct or indirect modulation of proteins associated with cell death.

133 Mutations in two PD genes, the E3-ligase Parkin and the deubiquitinating enzyme UCHL1, may lead to a susceptibility to UPS failure resulting in protein accumulation, Lewy body formation and dopaminergic cell death.

134 Furthermore, dysfunctional ?-synuclein and ?-synuclein oligomeric species have also been implicated in the impairment of the proteasome system [48], which in turn has been implicated in ?-synuclein turnover [15].

135 Several ubiquitin specific proteases (e.g. USP24 and USP40) have been consistently associated with PD [49]?[52].

136 To our knowledge, this is the first global miRNAs expression analysis performed in PBMCs in a relatively large cohort of PD patients and controls.

137 Taken together, this work suggests that a small subset of miRNAs may act as regulators of cellular mechanisms leading to PD.

138 Some of the pathways highlighted here are already known to be involved in PD pathogenesis, but others constitute new avenues for future research.

139 MATERIALS AND METHODS.

140 ETHICS STATEMENT.

141 The Lisbon University Hospital's ethical committee approved the study, and all participants or their legal representatives signed an informed consent.

142 PATIENT AND CONTROL SAMPLES.

143 Study participants were recruited at the movement disorders outpatient clinic of the Lisbon University Hospital over a period of eight months.

144 Patients were evaluated by neurologists with expertise in PD.

145 Diagnosis of Parkinson's disease was based on the UK Parkinson's Disease Society Brain Bank's criteria [53].

146 All recruited patients had more than two years of disease duration, therefore significantly reducing the probability of including atypical or secondary causes of parkinsonism [53].

147 Controls demonstrated no signs of parkinsonism and had no family history of PD.

148 Patients' clinical assessment included the modified Hoehn and Yahr staging [54], the Schwab and England Activities of Daily Living Scale [55], and the Unified Parkinson's Disease Rating Scale (UPDRS) [56].

149 All participants were Portuguese Caucasians.

150 SAMPLE COLLECTION AND RNA ISOLATION.

151 Approximately 16 ml of whole blood was collected per participant by venipuncture into two BD Vacutainer CPT glass tubes (Becton-Dickinson).

152 Within two hours of blood collection, PBMCs were isolated from the CPT tubes by centrifugation (30 minutes, 1639 g, room temperature), washed twice with PBS (15 minutes, 353 g, 4?C) and conserved on RNA later (Qiagen).

153 Total RNA containing small RNAs species was extracted from PBMCs using the miRNeasy Mini kit (Qiagen) according to the manufacturer's instructions, aliquoted and stored at ?80?C.

154 Since high-quality RNA is an absolute prerequisite to obtain reliable and reproducible microarray data, all samples passed an extensive quality control (e.g. OD260/OD280 between 1.8 and 2.1 in the Nanodrop ND1000 and RNA Integrity Number>7 in the Agilent 2100 Bioanalyser).

155 MIRNA PROFILING.

156 RNA samples were sent to Exiqon (Denmark) for miRNA profiling.

157 Briefly, 1 ?g of total RNA from samples meeting the quality standards described above were labeled with the miRCURY?

158 Hy3?/Hy5?

159 power labeling kit and hybridized on the miRCURY?

160 locked nucleic acid (LNA) array (version 10.0) using a Tecan HS4800 hybridization station.

161 Each sample was co-hybridized with a Hy5-labeled common reference pool composed of equal RNA concentration of the 39 samples (the 32 under study and 7 additional samples, later excluded from differential expression analysis due to array quality control problems).

162 Arrays were scanned with an Agilent G2565BA Microarray Scanner System and stored in an ozone-free environment in order to prevent potential bleaching of the fluorescent dyes.

163 Image analysis was carried out using the ImaGene 7.0 software (BioDiscovery) and the resulting ImaGene output files were analyzed using the Limma package [16] implemented in the R freeware (http://cran.r-project.org/).

164 Microarray data submission for human arrays is MIAME compliant.

165 Raw data from the microarray study have been deposited in the Gene Expression Omnibus (GEO) database with the accession number GSE16658.

166 Background correction was performed using the normexp method (with offset?=?10).

167 Intensities of good quality spots were then subjected to within-array (loess method) normalization.

[illegible]

168 Intensities lower than 1.5 times the median signal intensity of the respective slide and label were discarded.

169 The ?calling? of a miRNA on a particular array fails if two or more of the four replicated capture probes for this miRNA are flagged as bad quality spots by the image analysis software.

170 Differential miRNA expression between controls and PD patients was assessed using linear regression analysis and empirical Bayes methods implemented in the Limma package.

171 Hierarchical clustering and principal component analysis of the samples were performed with the Partek Genomics Suite software v.6.5 using the log2(sample/reference) ratios of the 18 differentially expressed miRNAs as input.

172 HC was performed using the Euclidean distance function and the average linkage clustering methods.

173 PCA analysis was set to run using the following options: covariance dispersion matrix (used when the variables are measured in the same units and have similar variances) and normalized eigenvector scaling (orthogonal eigenvectors and scaled to unit).

174 CHROMATIN IMMUNOPRECIPITATION FOLLOWED BY SEQUENCING ANALYSIS.

175 Human H4 neuroglioma cells (HTB-148-ATCC, Manassas) were maintained in OPTI-MEM (Life Technologies) supplemented with 10% fetal bovine serum and maintained at 5% CO2, 37?C to confluence.

176 The cells were cross-linked in 1% formaldehyde for 10 minutes at room temperature and pelleted by low-speed centrifugation (1000 g).

177 The cells were then lysed in lysis buffer (1.0% SDS, 10 mM EDTA, 50 mM Tris-HCl pH 8.1) containing Protease Inhibitor Cocktail (Roche).

178 Chromatin was sheared to an average DNA fragment size of 100?500 bp by sonication using the Diagenode Bioruptor sonicator (2?15 minutes of 30-second bursts at high output power with 30-second rest intervals between pulses) and fragment sizes were checked by agarose gel electrophoresis and with the Agilent Bioanalyzer DNA 1000 kit.

179 100 ?l of chromatin was aliquoted as total input (Input) and immunoprecipitated (IP) for later steps.

180 Chromatin was precleared with 45 ?l Protein G-Agarose solution (Upstate), followed by the immunoprecipitation using 2.5 ?g of ?-synuclein antibody (Ref. No. 610786, BD Biosciences) overnight at 4?C with slow rotation.

181 Subsequently, the beads were washed using Buffer 1 (0.1% SDS, 1% Triton X-100, 2 mM EDTA, 20 mM Tris-HCl pH 8.1, 100 mM NaCl), Buffer 2 (Buffer 1 with 500 mM NaCl), Buffer 3 (0.25 M LiCl, 1% NP-40, 1% C24H39NaO4, 1 mM EDTA, 10 mM Tris-HCl), and 2?

182 TE Buffer.

183 Chromatin was eluted by vortexing and rotation at room temperature in 1% SDS and 0.1 M NaHCO3.

184 To reverse the crosslinks, precipitated chromatin was incubated at 65?C overnight by adding NaCl (to 0.2 M) and then phenol-extracted.

185 The DNA was purified using the QIAquick PCR Purification Kit (Qiagen).

186 The total amount of DNA was measured using PicoGreen (Invitrogen) and a NanoDrop Spectrophotometer.

187 Immunoprecipitated DNA and total input DNA were blunt ended and adapters were ligated to the ends, according to the library preparation protocol from Illumina.

188 The DNA fragments with 200?25 bp in length were then selected for the construction of the ChIP-seq DNA library.

189 The quality of the libraries was checked using the Agilent Bioanalyzer HS DNA Kit, showing correct size and concentration of the samples.

190 Following the size selection, all the resulting immunoprecipitated DNA fragments were amplified and sequenced simultaneously using the Illumina Genome Analyzer II.

191 Using the ELAND software (Illumina), 9.694.993 reads were aligned to the repeat masked NCBI 36/hg18 build of the human genome, allowing up to two mismatches.

192 The Partek Genomics Suite (Partek) ChIP-seq analysis workflow was utilized to identify ?-synuclein enriched regions.

193 The software uses a sliding window approach to identify regions of higher read depth, referred to as peaks, relative to a background distribution.

194 We used a 100 bp window and the zero-truncated binomial model with a peak cut-off p-value of 0.001.

195 VALIDATION OF CHIP-SEQ RESULTS BY CHIP REAL-TIME PCR.

196 The original IP and Input DNA aliquots from the microarray experiment were used to assess the quality of the ChIP.

197 ?-synuclein occupancy was detected by qPCR at the CDC-42 and NEDD4 promoters.

198 Primers were designed with the PRIMER3 program (http://frodo.wi.mit.edu/cgi-bin/primer3/primer3-www.cgi).

199 The primer sequences were as follows: CDC-42 forward GATGCACCACTGTTCTCCAG, reverse AGACACCTCTCCTCCTTCCAC; NEDD4 forward GTAACGAGCCCTTCCTGTGA, reverse TCAACTACCCGACCGACTTC; myoglobin exon 2 forward AAGTTTGACAAGTTCAAGCACCTG, reverse TGGCACCATGCTTCTTTAAGTC.

200 The qPCR was performed on a Roche LightCycler in triplicate.

201 The quality of the ChIP was calculated as fold enrichment over myoglobin exon 2 non-binding control region.

202 PATHWAY ANALYSES.

203 Predicted target genes or the ?-synuclein interacting genes were uploaded into the commercially available IPA software v.7.6 for biological function and pathway analysis.

204 IPA is built upon a very large manually-curated and up-to-date database of genes, proteins, functions, interactions/networks, and pathways.

205 The IPA ?Core Analysis?

206 was performed using the Ingenuity Knowledge Base (IPAKB, Genes only) as the reference set, using direct and indirect relationships for network analysis, and data from mammal species, and all tissues, cell lines and data sources.

207 The significance (p-values) of the association between a dataset and a canonical pathway was determined by comparing the number of genes in a dataset that participate in a given pathway to the total number of occurrences of these genes in all pathway annotations that are stored in the IPAKB.

208 A Fisher's exact test was used to calculate the p-value to determine the probability that the association between the genes in the dataset and the canonical pathway is explained only by chance.

209 The level of statistical significance was set to $p<0.05$.

210 Each pathway analysis generated the top canonical pathways with a statistical significance (Figures S4A and S4B).

211 WHOLE-GENOME ASSOCIATION DATA MINING.

212 A joint association analysis was performed on three PD GWAS datasets: The HIHG at the University of Miami [13], the NINDS [9], and the joint dataset from the Progeni/GenePD studies that was genotyped at the CIDR [10].

213 This meta-dataset has a combined sample size of 1752 cases and 1745 controls.

214 Details regarding the characteristics of the study participants and the markers analyzed in each dataset are described in detail in the original reports [9], [10], [13].

215 Briefly, genotype and phenotype data from the NINDS and Progeni/GenePD studies were downloaded from dbGAP (http://www.ncbi.nlm.nih.gov/sites/entrez?db=gap).

216 HIHG genotype data were generated using the Illumina Infinium 610-quad BeadChip.

217 Imputation of SNP genotypes from the three GWAS dataset was performed using the software package Impute [57].

218 Samples with a genotyping efficiency <0.98 and SNPs with a genotyping call rate <0.98 were removed from the analysis.

219 In addition, SNPs with a minor allele frequency <0.01 or a Hardy-Weinberg equilibrium p-value<10?7 (in controls) were excluded.

220 Population stratification was evaluated using Eigenstrat [58].

221 Cochran-Armitage trend tests were used to assess allelic association at each SNP using PLINK [59].

222 SUPPORTING INFORMATION.

223 Figure S1Validation of microarray expression results by qRT-PCR of five miRNAs.

224 Five miRNAs showing distinctive expression patterns between PD and control samples were selected for global validation of the microarray results by qRT-PCR on the same dataset of 19 PD cases and 13 controls.

225 We selected miRNAs (miR-126*, miR-32 and miR-101) that were under-expressed (fold-change <?1), and miRNAs (miR-15b and miR-550) that were over-expressed (fold-change>1) in the microarrays, both above (miR-126*) and below (miR-32, miR-101, miR-15b, and miR-550) our threshold for significance (B-statistic?1).

226 Normalization to endogenous expression levels of control genes is currently the most accurate method to correct for potential RNA input or RT efficiency biases.

227 The NormFinder algorithm [61] was used to assess the variance in expression levels in our miRNAs and to select the best candidate endogenous controls (three small nuclear/nucleolar RNAs RNU66, RNU6B and Z30, and miRNA miR-103) for qPCR normalization.

228 Quantification of selected miRNAs and endogenous controls by TaqMan?

229 Real-Time PCR was carried out as described by the manufacturer (Applied Biosystems [ABI]).
230 Briefly, 50 ng of template RNA was reverse transcribed using the TaqMan?
231 MicroRNA Reverse Transcription Kit and stem-loop miRNA-specific primers from the ABI assays (TaqMan? MiRNA assays miR-126*, miR-32, miR-101, miR-15b, miR-550, miR-103, RNU66, RNU6B and Z30).
232 1.33 ?l of each RT product was PCR-amplified in triplicate in 20 ?l PCR reactions (95?C for 10 minutes, followed by 50 cycles of 95?C for 15 seconds and 60?C for 1 minute) in 384-well plates on the ABI 7900HT Fast Real-Time PCR System (ABI).
233 For each assay, a paired no-template control (NTC) and a common reference pool (equal quantity of RNA from the 19 cases and 13 controls) reactions were performed.
234 Based on the qRT-PCR results, miR-103 was selected by NormFinder as the best endogenous control (stability value of 0.223) and was further used as the reference miRNA to normalize expression levels.
235 Expression values were calculated using the ??CT method: 2?(?CT[sample]??CT[pool]) with ?CT?=?CT(selected miRNA)?CT(miR-103) [62].
236 The PD/control expression ratio (expected value between 0 and +?) is calculated by dividing the average ??CT of cases by the average ??CT of controls.
237 Ratio values between 0 and 1 indicate that a miRNA is down-regulated in PD patients, and values greater than 1 mean that it is up-regulated in PD patients.
238 Ratio values can be converted to the more intuitive fold-change (FC) values by taking the negative inverse (?1/ratio) for ratios between 0 and 1 (e.g. a ratio of 0.5 corresponds to a ?2 FC, which means that the miRNA is 2-fold down-regulated in cases).
239 FC values are equal to ratios for ratios greater than one.
240 The expected values of FC are]?
241 ?;?1] and [1;+?[.
242 A two-tailed t-test with unequal variance was used to assess significant differences in qRT-PCR among cases and controls.
243 The table below the graph indicates the fold-changes and B-statistics obtained for the selected miRNAs in the microarray experiment, as well as the fold-changes and p-values obtained in the qRT-PCR experiment.(PDF)Click here for additional data file.
244 Figure S2Two-dimensional principal component analysis (PCA) plot of the 32 individuals.
245 PCA analysis was based on the expression levels of the eighteen differentially expressed miRNAs.
246 Control and patient samples are shown in black and red, respectively.
247 Males are represented by squares and females by circles, and the individual's ID (Table 1) is indicated inside the symbol.(PDF)Click here for additional data file.
248 Figure S3Sources of variation in the miRNomics expression data.
249 Analysis of variance (3-way ANOVA) was used to evaluate the impact of several potential sources of variation (age, gender and affection status) in our expression data.(PDF)Click here for additional data file.
250 Figure S4Top ten canonical pathways over-represented among the predicted target genes of 11 out of the 18 differentially expressed miRNAs (A) and among the 238 genes targeted by ?-synuclein (B).
251 These analyses were carried out using the Ingenuity Pathway Analysis software and each plot displays the pathways ranked by significance level on the y-axis.
252 The x-axis on the top is for the negative logarithm of the p-value (orange dots connected by a line).
253 The significance (p-values) of the association between a dataset and a canonical pathway was determined by comparing the number of genes in a dataset that participate in a given pathway to the total number of occurrences of these genes in all pathway annotations that are stored in the Ingenuity Knowledge Base.
254 The p-value is calculated using the right-tailed Fisher exact test to determine the probability that the association between the genes in the dataset and the canonical pathway is explained only by chance.
255 The number of genes in our dataset in a given pathway and the total number of genes associated with that pathway in the IPA's database are shown to the right of each pathway and are used to calculate the percentage displayed in the grey bars (the x-axis on the bottom is for the grey bars).
256 The percentage and the significance are measures of the amount and confidence, respectively, of association of a given canonical pathway with the data.(PDF)Click here for additional data file.
257 Table S1959 target genes (662 unique genes) predicted by miRecords for 11 differentially expressed miRNAs.
258 Only genes that are predicted to be targets by at least six softwares are shown.(XLS)Click here for additional data file.
259 Table S2Top ten canonical pathways in the Ingenuity Pathway Analysis of the 662 genes predicted to be targets of 11 differentially expressed miRNAs.
260 The target genes and their respective miRNA in each pathway are indicated.
261 The ratio corresponds to the number of genes in a pathway that were in our target gene list divided by the total number of genes in that pathway.
262 The p-value is calculated using the right-tailed Fisher exact test and takes into account the number of genes in our dataset in a given pathway and the total number of genes associated with that function in IPA's database.
263 The ratio and the significance are measures of the amount and confidence, respectively, of association of a given canonical pathway with the data.(XLS)Click here for additional data file.
264 Table S3ChIP-seq target genes containing the ?-synuclein enriched regions overlapping with the transcribed sequences.(XLS)Click here for additional data file.
265 Table S4Allelic association results for SNPs in the glycosphingolipid biosynthesis - ganglioseries and the protein ubiquitination pathway target genes.(XLS)Click here for additional data file.
